# From intersectional position to migrant standpoint: Ghanaian migrants’ knowledges of wellbeing, care, and constraint in Alberta, Canada

**DOI:** 10.3389/fsoc.2026.1914141

**Published:** 2026-09-30

**Authors:** Noah Boakye-Yiadom, Isaac Nortey Darko, Bukola Salami

**Affiliations:** 1 University of Calgary Cumming School of Medicine, Calgary, AB, Canada; 2 Department of Sociology, Algoma University, Sault Ste. Marie, ON, Canada

**Keywords:** African migrants, health equity, indigenous methodologies, intersectionality, mental health promotion, migrant standpoint, Ori-Oyena, standpoint theory

## Abstract

**Introduction:**

Intersectionality has strengthened migration scholarship by showing how race, gender, class, legal status, culture, and other relations of power shape migrant lives. However, it has been less explicit about how migration-shaped locations become sites of interpretation, critique, and knowledge production. This article addresses that gap by developing the concept of migrant standpoint through the case of mental health promotion among Ghanaians in Alberta, Canada.

**Methods:**

The study used a qualitative design grounded in Ori-Oyena, an African-grounded methodological orientation centered on relational knowledge, story, participation, and communal interpretation. Data were generated through in-depth interviews with Ghanaian adults in Alberta and a community dialogue with leaders that refined emergent interpretations. Analysis proceeded iteratively, combining qualitative coding with interpretive attention to how participants defined wellbeing, explained constraint, and described the conditions under which support became possible or difficult to sustain. Participants varied by gender, migration pathway, occupational location, religious affiliation, and length of residence, and the analysis treats that variation as constitutive of standpoint rather than as background noise.

**Results:**

Participants understood wellbeing in relational rather than narrowly individual terms, linking it to spiritual grounding, family relationships, moral responsibility, and social legitimacy. Four interrelated dimensions of migrant standpoint emerged: relational wellbeing; translated survival knowledge; institutional misrecognition and selective engagement; and double vision as comparative social knowledge. Together, these findings showed that migration shaped not only exposure to inequality but also the interpretation of care, distress, and institutional fit. These dimensions were internally differentiated: religious practice supplied a rival system for authoritatively naming distress, and the relational labour on which wellbeing depended was gendered, falling disproportionately on women whose kin networks had not migrated with them.

**Discussion:**

Migrant standpoint extends intersectional migration scholarship by shifting attention from social position alone to socially situated knowledge shaped by movement, settlement, racialization, and institutional negotiation. Mental health promotion proved to be a revealing analytic site because it exposed broader struggles over recognition, belonging, legitimacy, and the conditions under which wellbeing can be sustained. Rather than proposing a universal migrant standpoint, this article shows how a bounded empirical case can clarify a broader sociological mechanism through which migrant communities generate knowledge under altered structural conditions.

## Introduction

Intersectionality plays a crucial role in migration scholarship by highlighting that the experiences of migrants are not defined by just one factor, but rather by the complex interplay of race, gender, class, nationality, legal status, religion, and other power dynamics (Anthias, 2008; Cleton and Meier, 2023; Collins, 2010; Yuval-Davis, 2006). This body of work has been especially important in demonstrating that migrant experiences of belonging, exclusion, precarity, and recognition are internally differentiated and historically produced, and not uniform across or within groups (Anthias, 2002, 2008; Yuval-Davis, 2006). However, an important gap remains. While intersectional migration scholarship has become increasingly sophisticated in mapping social location and unequal incorporation, it has been less explicit in theorizing what forms of knowledge become possible from those locations, particularly under conditions shaped by migration, racialization, settlement, and institutional negotiation (Amelina, 2022; Amelung et al., 2024). This article addresses this gap by advancing the concept of migrant standpoint as a way of bringing questions of socially situated knowledge more directly into migration research.

Drawing on qualitative research with Ghanaian migrants living in Alberta, Canada, this article argues that migration does more than add another layer of inequality to intersectional analysis. Migration can also produce a distinct socially situated mode of knowing, formed through transnational movement, racialized incorporation, shifting obligations to family and community, and repeated encounters with institutions that do not fully recognize migrants’ own frameworks of wellbeing, care, and social life (Anthias, 2008; Yuval-Davis, 2006). Using mental health promotion as the empirical lens, we show how participants interpret wellbeing, distress, support, and structural constraints from positions shaped not only by who they are, but also by what migration requires them to navigate, preserve, translate, and resist. By doing so, this paper establishes the migrant standpoint as a theoretically valuable extension of intersectional analysis, shifting focus from position alone to the interpretation and critique made possible by migration-shaped experiences (Amelina, 2022; Amelung et al., 2024; Anthias, 2008). Because intersectionality is an empirical as well as a theoretical commitment, the analysis that follows does not treat “Ghanaian migrants” as a single, undifferentiated group. It traces how gender, migration pathway, class and occupational location, religious affiliation, and length of residence produce different vantage points within a shared community, and therefore different, and sometimes competing, migrant standpoints. Religion is given particular analytic weight, since spiritual practice and church membership emerged in participants’ accounts not as background colour but as an infrastructure through which wellbeing was defined, distress was interpreted, and formal services were assessed (Collins, 2010; Yuval-Davis, 2006).

### Intersectionality, standpoint, and migration

Intersectionality has become one of the most influential frameworks for understanding migrant lives because it explains how multiple relations of power, including race, gender, class, nationality, religion, and legal status, shape the experiences of mobility, settlement, and exclusion in mutually constitutive ways (Anthias, 2002). Rather than treating inequality as the product of a single axis, intersectional scholarship has shown that migrant experiences are shaped by overlapping, historically situated structures that distribute vulnerability, recognition, and opportunity unevenly across social groups (Anthias, 2008; Yuval-Davis, 2006). In migration studies, this perspective has been particularly useful for challenging flattening accounts of “the migrant” as a uniform category and demonstrating that social location is always differentiated within migrant populations (Anthias, 2008). While this body of work has significantly sharpened analyses of stratification and belonging, it has been less explicit about what these locations make possible in epistemic terms, that is, how migrants come to know, interpret, and critique social life from within migration-shaped conditions.

Social location refers to where people are positioned within historically produced relations of power, including race, gender, class, migration status, generation, and religion. It identifies the structural coordinates through which inequality is organized and lived (Anthias, 2008; Harding, 1996). Standpoint, by contrast, refers to the socially situated way of knowing that may emerge from living, interpreting, and reflecting on those positions over time. A shared location does not automatically produce a shared standpoint. Rather, standpoint is better understood as an achieved interpretive and epistemic orientation, shaped through experience, comparison, and engagement with power-laden social worlds (Collins, 2000; Harding, 1996; Rolin, 2009). In this article, migration is treated not only as a social location within unequal structures, but also as a constitutive process through which a standpoint may emerge, as people learn to interpret social life across different moral, cultural, and institutional worlds (Anthias, 2008).

Standpoint theory offers an important conceptual resource for addressing these limitations. Emerging from feminist epistemology and sociology, standpoint theory argues that knowledge is socially situated and that marginalized social locations can provide distinctive insights into the organization of power precisely because those subject to domination must navigate social worlds that were not built around their ease or visibility (Harding, 1996; Hartsock, 1983). However, standpoint theory does not reduce knowledge to experience. Its strongest formulations insist that a standpoint is not automatically possessed by virtue of social position, but is achieved through reflection, struggle, and the collective interpretation of lived contradictions (Haraway, 1988; Harding, 1996; Rolin, 2009). This distinction between location and standpoint is central to the present study. This allows migration to be understood not merely as a demographic characteristic or an axis of disadvantage but as a social process through which particular ways of knowing may be formed.

Foundational standpoint theorists provide a basis for this move. Hartsock (1983) argued that subordinated positions can generate a critical angle on dominant social arrangements, while Harding (1996) extended this argument through the idea of strong objectivity, contending that inquiry that begins from marginalized lives may produce more robust accounts of social reality than inquiry that leaves dominant assumptions unexamined. Haraway (1988) similarly rejected both detached universalism and uncritical relativism by insisting that all knowledge is partial, embodied, and accountable to the position from which it was produced. In sociology, Smith (1987, 1992) emphasized that inquiry should begin from people’s everyday worlds rather than abstract institutional categories that obscure lived social relations. In the tradition of institutional ethnography that develops from this position, standpoint is not only a situated way of seeing but an empirical point of entry: beginning from the everyday world of those outside the centres of institutional power allows inquiry to trace how translocal institutional relations organize local experience, often in ways that remain invisible to the institutions themselves (Smith, 1987, 1992). Collectively, these scholars elucidate that standpoint theory extends beyond merely identifying those who are oppressed. It is about how social location influences our perceptions, shaping what we are able to see, name, and challenge. This is especially relevant for migration research, where movement across social worlds may intensify the visibility of institutional assumptions, normative orders, and misalignments that remain less visible to those more fully centered within dominant settings.

This gap matters because migration does not simply place people into pre-existing structures of difference. It also reorganizes how these structures are lived, compared, and understood. Migrants often navigate more than one moral order, framework of belonging, and set of institutional expectations simultaneously (Smith, 1987, 1992). As a result, they may develop forms of comparative and critical perceptions that are not reducible to an identity category alone. However, much of the existing literature still stops at the description of social position, even when it is theoretically sophisticated in other respects. In other words, the literature has strongly shown that migrant lives are intersectionally structured, but less developed in explaining how migration-shaped positions become sites of knowledge production. This is where the concept of migrant standpoint becomes useful. It shifts attention from layered disadvantage alone to the interpretive, moral, and analytic capacities formed through movement, racialization, settlement, and negotiation with institutions.

### Toward a concept of migrant standpoint

Although migration scholarship rarely consolidates these insights under the formal label of “migrant standpoint theory,” several adjacent literatures point clearly in that direction. Collins (1986, 2000) concept of the outsider within is particularly important because it theorizes how marginal yet proximate locations can generate critical knowledge of institutions, norms, and social contradictions. While Collins developed this concept in relation to Black feminist thought, its analytic relevance extends to migrant contexts in which individuals are required to participate in institutions that do not fully recognize or reflect their social realities. Similarly, Anthias (2002, 2008) work on translocational positionality has shown that identity and belonging in migration contexts are not fixed but are continuously shaped across shifting relations of time, place, and power. Yuval-Davis (2006) adds to this by arguing that belonging is not merely an emotional matter but is tied to political and social struggles over who is recognized, valued, and authorized to inhabit space. These contributions suggest that migrants do not simply experience layered identities. They occupy shifting, relational, and often contradictory positions from which comparative social knowledge emerges.

Recent migration scholarship has pushed this issue further by turning its attention to the politics of knowledge production itself. Rather than treating migrants primarily as subjects to be described, measured, or integrated, scholars have increasingly asked who gets to produce knowledge about migration, which standpoints shape dominant interpretations, and how colonial and hegemonic assumptions continue to organize research agendas in the field (Amelina, 2022; Amelung et al., 2024). This literature is especially important because it reframes migration research as an epistemic and empirical problem. It asks not only what migrants experience but also how migration studies have often failed to take migrants seriously as knowers of the social worlds they inhabit. Related methodological work on insider, outsider, and reflexive positionality in diasporic research reinforces this shift by showing that migrant and diasporic locations are not incidental to inquiry but can be central to how meaning is accessed, interpreted and contested (Ademolu, 2024). However, this emerging scholarship has not yet fully theorized the migrant standpoint as a distinct analytic concept linked to everyday life, institutional encounters, and the production of practical and critical knowledge through migration.

### Mental health promotion as an analytic site

Mental health promotion is a valuable area for understanding migrants’ perspectives because it lies at the intersection of daily life, social interactions, institutional frameworks, and the broader conditions that determine whether life is worth living (Barry et al., 2023; Compton and Shim, 2015). Contemporary public mental health literature defines mental health as more than the absence of disorder and emphasizes that promotion depends on individual, social, and structural determinants, including social connection, education, housing, work, safety, and community environment (Compton and Shim, 2015; Kirkbride et al., 2024). This broader framing is especially important in migration contexts, where wellbeing is often shaped by disrupted support networks, altered family and kinship roles, experiences of racialization, economic precarity, and the demands of adaptation within institutions that may not reflect migrants’ own understandings of care and social life (Jarvis and Kirmayer, 2023; Patel, 2005). Therefore, mental health promotion cannot be reduced to service uptake or prevention messaging alone. It also encompasses the routines, relationships, norms, and material conditions through which people sustain their dignity, hope, connectedness, and everyday balance.

This framing is particularly productive for the present study because it allows migrant wellbeing to be examined as a socially situated and culturally mediated practice rather than as an exclusively individual or psychological state. Emerging Canadian evidence indicates that among Black communities, positive mental health is often supported by spiritual resources, collective culture, resilience, and culturally congruent forms of support, while stigma, service inaccessibility, and poor cultural fit remain important barriers to care and wellbeing (Olanlesi-Aliu et al., 2025). Related work with migrant and diasporic communities likewise shows that institutional encounters are shaped by language, trust, cultural interpretation, and the extent to which formal systems can recognize migrants’ lived realities and preferred forms of support (Lay et al., 2024; Meribe et al., 2025). Examining mental health promotion through this lens makes the questions of recognition, translation, legitimacy, and structural fit visible. Therefore, mental health promotion is not only a public health concern but also the focus of this study. It is also a sociological analytic site through which the migrant standpoint can be developed and examined.

### Study context

The present article addresses this gap by drawing on qualitative research with Ghanaians living in Alberta to theorize migrant standpoint through the empirical site of mental health promotion. Mental health promotion is especially productive for this purpose because it reveals how wellbeing is understood, sustained, and contested within everyday social life rather than only within formal systems of diagnosis or treatment. It highlights the moral, relational, spiritual, and institutional dimensions through which migrants assess what it means to live well, what threatens that possibility, and which forms of support feel socially legitimate or culturally coherent. By using this empirical case to develop the concept, this article does not claim to identify a single or universal migrant standpoint. Rather, it argues that migration can generate socially situated ways of knowing that deserve more explicit theorization in sociology. In this sense, the paper’s contribution is both empirical and conceptual, using a bounded case to extend intersectional migration scholarship toward a fuller account of knowledge, standpoint, and social life under conditions of migration.

### Ghanaian migration and settlement in Alberta

Alberta is not simply where this study happened; it is a theoretically informative site. Ghanaian migration to Canada began in earnest in the 1960s and consolidated around Ontario, and the Greater Toronto Area in particular, which remains the country’s principal Ghanaian hub and the setting for most of the existing Canadian scholarship on Ghanaian associational life, remittance practice, and community formation (Owusu, 2000). Alberta’s Ghanaian population is smaller, younger, and more recently established. Alberta’s Ghanaian population is comparatively small but expanding rapidly. Residents reporting Ghanaian ethnic or cultural origin rose from 4,465 in 2016 to 6,255 in 2021, an increase of 40.1%. Over the same period Alberta’s total population grew by 4.8%, so the Ghanaian-origin population expanded at roughly eight times the provincial rate. This is a community whose growth is recent enough that its associational and institutional infrastructure is still being assembled rather than inherited (Statistics Canada, 2022). Its growth has been driven less by chain migration into a long-settled community than by labour-market recruitment into Alberta’s resource, health, and service sectors, by international study pathways, and by secondary migration from Ontario and Quebec in search of housing affordability and employment. Edmonton and Calgary now rank among Canada’s largest metropolitan concentrations of Black residents, and over half of Canada’s Black immigrants from Africa arrived in the decade to 2021 (Statistics Canada, 2022).

These conditions matter analytically rather than merely descriptively. Where Ontario offers a dense institutional infrastructure of Ghanaian churches, hometown associations, and ethnic media accumulated over six decades, Alberta offers a thinner and more improvised one. Community organizations are newer, geographically dispersed across two mid-sized cities and a large rural hinterland, and more dependent on individual effort to sustain. Alberta also combines relatively high incomes with a boom-and-bust resource economy, a provincial health system in which mental health services are unevenly distributed, and a political culture organized around individual self-sufficiency. The result is a setting in which the gap between relational expectations carried from Ghana and the institutional and social arrangements encountered in Canada is unusually exposed. Precisely because the communal scaffolding is not simply assumed to be there, participants had to articulate what it was for. Alberta therefore functions in this article as a case in which the interpretive labour that constitutes migrant standpoint becomes visible, rather than as a neutral container for Ghanaian experience (Salami et al., 2019).

## Methods

### Methodological orientation

This study was grounded in Ori-Oyena, an African-grounded methodological orientation developed to centre relational knowledge, story, participation, and communal interpretation (Boakye-Yiadom et al., 2026). Ori-Oyena aligns with broader African-centred and Indigenous methodological scholarship that treats knowledge as socially produced, ethically mediated, and culturally situated rather than as abstract information extracted from isolated individuals (Ahmed and Gyamerah, 2025; Chilisa, 2020; Mbah et al., 2024). This approach was particularly well-suited to the present study because the analysis focused not only on what participants experienced but also on how they interpreted those experiences and how meaning was refined in relation to shared social worlds (Boakye-Yiadom et al., 2026). In this sense, the approach rejects detached universalism and instead privileges socially located interpretation, a stance that is also consistent with standpoint theory’s insistence that knowledge emerges from historically and socially situated positions rather than from a view from nowhere (Harding, 1996; Rolin, 2009).

Ori-Oyena is grounded in the ontology of the Nafaanra (Nafana), an Indigenous West African people of northwestern Ghana around Sampa and Banda, whose Senufo language and proverb-rich oral traditions are closely linked through trade and intermarriage with neighbouring Akan communities (Boakye-Yiadom et al., 2026). Naming this provenance matters, because African-grounded methodologies are too often invoked as though the continent held a single epistemology. It does not, and Ori-Oyena is offered as a located Nafaanra and Akan method rather than a generic African one. Its morphemes carry specific senses: Ori denotes an autonomous yet interdependent “we,” and Oyena denotes seeing and verifying with one another, so that the name itself encodes the method’s commitment to mutual validation rather than extraction. The orientation rests on the axiom of simultaneous multiplicity, in which the observable and the spiritual are treated as co-empirical rather than as fact and belief, which is why participants’ spiritual accounts of wellbeing are analysed here as knowledge claims rather than as culture to be noted.

The full methodology, its ontological and axiological foundations, its five-phase procedure, and its braided analytic protocol, is set out in detail elsewhere and is summarised here only as it bears on this study (Boakye-Yiadom et al., 2026). Rather than claim novelty by renaming procedure, Ori-Oyena operationalises relational commitments that African-centred scholarship has long affirmed, extending Chilisa’s postcolonial Indigenous paradigm into an auditable sequence and answering, in Nyamnjoh’s convivial spirit, the charge that inherited frameworks presume their own completeness (Chilisa, 2020; Nyamnjoh, 2017). It also takes seriously Táíwò’s (2022) warning against a nativism that prizes whatever can be presented as indigenous: institutional consent, written documentation, and external peer scrutiny are retained here alongside locally recognised rites, and the warrant for the method is not that its practices are African but that they leave an inspectable trace from field encounter to community-validated theme.

Three features of that procedure did analytic work in this study that a conventional qualitative design would not have done. First, Ori-Lefuor, the entry phase, secured consent through a shared water rite conducted with community leaders and elders at each event, alongside institutional written consent, so that agreement was collective and renewed rather than transacted once with an individual. This is why participants spoke about family shame, church standing, and community reputation as freely as they did: those relationships were treated as the social units within which wellbeing is decided rather than as confidentiality risks to be managed, and the relational account of wellbeing reported below follows directly. Second, Ori-Ojawa, the storied and proverbial exchange, licensed participants to reason in narrative, proverb, and Twi rather than in the clinical register that mental health interviews typically impose; the proverb offered by EA below is not decorative but the participant’s own claim about interdependence, which a symptom-oriented schedule would have foreclosed. Third, Ori-Odorta returned the emerging themes to two community mapping circles for validation and, where needed, root-realignment, so that interpretation was checked by the people whose lives were described. Read through this procedure, institutional misrecognition emerged as a problem of interpretive authority rather than of service access, which is the reading the mapping circles produced.

The broader study included in-depth qualitative interviews with Ghanaian adults living in Alberta and community dialogue with leaders that served as a forum for refining emergent interpretations. Participants represented diverse migration histories and social positions, enabling attention to variation within the Ghanaian community while also identifying recurring patterns relevant to theorizing migrant standpoint. The analysis combined standard qualitative procedures with interpretive attention to how participants defined wellbeing, explained constraints, and described the conditions under which support became possible or difficult to sustain. This strategy is consistent with contemporary qualitative guidance that emphasizes trustworthiness through reflexive, relational, and dialogic forms of interpretation rather than through claims of neutrality alone (Ahmed, 2024; Braun and Clarke, 2025; Salzano et al., 2023). The aim was not to produce a universal theory of migrant life but to develop a concept from a bounded empirical case in a way that remained analytically disciplined and theoretically generative (DeMatthews et al., 2026).

### Data collection and analysis

Data for this article were derived from twenty-one (21) in-depth interviews with Ghanaian adults living in Alberta and one community dialogue involving eleven (11) participants with leadership roles or extensive community knowledge. Participants were purposively recruited to reflect a range of migration histories, settlement trajectories, and social positions relevant to the study’s focus on wellbeing and mental health promotion. In keeping with qualitative approaches designed to generate rich, contextually grounded accounts of lived experiences, the interviews invited participants to reflect on how they understood mental health and wellbeing, what practices sustained them, what pressures strained their lives, and how migration and settlement shaped the possibilities for care, support, and social balance (Lim, 2025). The community dialogue functioned not as a simple confirmatory exercise but as a forum for deepening, challenging, and refining emergent interpretations through collective reflection, an approach consistent with dialogic and community-engaged strategies for strengthening qualitative trustworthiness (Boakye-Yiadom et al., 2026; Salzano et al., 2023). Interviews were conducted in English and Twi according to participant preference, and several moved fluidly between the two; the first author, who is fluent in Twi, conducted these directly, so that proverbial and idiomatic speech was interpreted in place rather than through a third-party translator. Recordings were transcribed verbatim, with Twi-language passages translated by the first author and checked for meaning against the audio rather than rendered word for word. Given the close-knit character of the Ghanaian community in Alberta, confidentiality required more than the removal of names: potentially identifying details of occupation, congregation, and family circumstance were generalized in reporting, participant identifiers carry only the attributes relevant to the interpretation at hand, and the fuller procedural account of consent and data protection under the Ori-Oyena framework is set out in the methodological paper on which this study draws (Boakye-Yiadom et al., 2026). Community dialogue participants were selected for recognized standing and breadth of community knowledge across associational, religious, and service domains rather than as a representative panel, and the dialogue was treated as a site of contestation rather than consensus. Where leaders disagreed with an emergent interpretation, or with one another, the divergence was retained in the analysis rather than resolved by majority, and interpretations that less institutionally connected interviewees had voiced were weighed against, not subordinated to, leadership readings; the aim was to keep visible the internally differentiated and sometimes competing standpoints the article describes.

The analysis proceeded iteratively and interpretively. Interview transcripts and community dialogue materials were read multiple times to identify recurring concepts, tensions, and relational patterns, with coding focused on understandings of wellbeing, promotion practices, institutional encounters, moral obligations, social pressures, and transnational or comparative reference points. These codes were then grouped into higher-order analytic categories that spoke directly to the paper’s theoretical aim, moving from patterned meanings toward a conceptually organized account of migrant standpoint (Braun and Clarke, 2025; Naeem et al., 2023). The analysis did not treat neutrality as a benchmark of rigour but emphasized reflexive, relational, and dialogic interpretation (Ahmed S. K., 2024; Braun and Clarke, 2025). Rather than presenting themes as descriptive findings alone, the analysis asked how participants’ accounts revealed distinct dimensions of migrant standpoint and, in doing so, supported a sociological argument about migration-shaped ways of knowing. Because insider familiarity can naturalize precisely what analysis needs to make strange, coding proceeded by asking of each apparently obvious meaning what a participant was claiming and on what grounds, rather than assuming a shared reading; interpretations were tested against the community dialogue, and accounts that ran counter to an emerging pattern were treated as occasions to qualify or bound the claim rather than as noise. The patterned differences reported below by gender, migration pathway, occupation, religion, and length of residence were retained as analytic claims only where they held across multiple accounts and where divergent cases could be accommodated or explained, and they are stated as tendencies within a small qualitative sample rather than as distributions.

### Participants

Twenty-one adults who were born in Ghana and living in Alberta took part in individual interviews, and eleven community leaders took part in the dialogue. Because the analytic claim of this article is intersectional, sample composition is reported in detail rather than summarized as diversity in the abstract (see Table 1).

**Table 1:** Participant sociodemographic characteristics (*N* = 21).

Characteristic	*n* (%) or Mean ± SD
Age, years	35.3 ± 10.6
Age range	22 to 58
Gender	
Male	9 (42.9)
Female	9 (42.9)
Prefer not to say	3 (14.3)
Relationship status	
Single	8 (38.1)
Married	7 (33.3)
Widowed	3 (14.3)
Missing	3 (14.3)
Educational status	
University	12 (57.1)
College	3 (14.3)
Diploma or technical program	1 (4.8)
Other	1 (4.8)
Prefer not to answer	1 (4.8)
Missing	3 (14.3)
Ethnic background	
Akan	13 (61.9)
Ewe	2 (9.5)
Ga-Adangbe	2 (9.5)
Dagaaba/Waala	1 (4.8)
Missing	3 (14.3)
Years in Canada	
Canadian-born	3 (14.3)
<2 years	5 (23.8)
2–9 years	4 (19.0)
≥10 years	7 (33.3)
Missing	2 (9.5)

Percentages are calculated using the full interview sample (*N* = 21). Age was reported by 17 participants; gender, relationship status, educational status, and ethnic background by 18 participants; and migration information by 19 participants. Missing denotes item non-response. Years in Canada was grouped from self-reported responses as Canadian-born, less than 2 years, 2–9 years, or 10 years or more.

Participant identifiers used throughout the findings carry the attributes relevant to the interpretation being made, so that readers can see which position a given claim is being made from.

### Positionality

A study that argues knowledge is always situated cannot exempt the knowledge it produces from that claim. As first author, I conducted all interviews and facilitated the community dialogue, and I write from a particular location. I am a Ghanaian-born immigrant who came to Canada as a teenager, the child of a Nafaanra mother, an ethnically marginalized people whose territory straddles Ghana and Côte d’Ivoire, and a Bono (Akan) father. My family arrived through political asylum, reuniting in Toronto in the late 1990s after a seven-year separation, and I grew up in Rexdale, a Greater Toronto Area priority neighbourhood, within a Ghanaian Seventh-day Adventist congregation and the Ghanaian associational life around it. I speak Twi and my father’s Bono dialect, and portions of the storied and proverbial exchanges were conducted in Twi, allowing participants to reason in proverb and idiom without translating themselves for an outsider. My upbringing in Adventist Christianity, alongside an extended family that includes Muslim, Catholic, and Traditionalist relatives, shaped how participants spoke to me about faith, affording a shared vocabulary for spiritual accounts of wellbeing while also marking me as situated within, rather than neutral toward, the religious landscape I was studying. I have been a member of the Ghanaian-Canadian associations of Ontario, Calgary, and Red Deer, and worked for over 5 years as a health promotion facilitator with Alberta Health Services, so recruitment through Ghanaian associations and churches proceeded through networks to which I already belonged. At the time of the study, I was a doctoral researcher at the University of Calgary, an institutional position that made me legible to some participants as an emissary of the very systems whose recognition they were seeking or contesting. The co-authors bring positions that bear on the analysis, including supervisory expertise in immigrant and refugee health and in African-centred research.

Insider status was partial and unevenly distributed across the sample, and it cut both ways. Shared origin, language, and idiom made certain things speakable quickly: participants used proverbs without translating them, named spiritual causation without hedging, and criticized Canadian institutions without the diplomatic softening that Black participants often perform for white researchers. It also made certain things harder to say. Where I was legible as a member of the same community, and therefore as someone who might encounter a participant again at church or at a funeral, disclosures about marital difficulty, psychiatric contact, financial failure, or immigration precarity carried reputational risk that they would not have carried with a stranger. Several participants managed this by speaking in the third person about “some people in the community,” a move that was itself analytically informative. Insider access, in other words, did not produce unmediated truth; it produced a particular distribution of candour and reticence, and I have tried to read that distribution rather than look through it (Ademolu, 2024; Akuffo, 2023).

My university affiliation introduced a second asymmetry. To participants who had experienced institutional misrecognition, I was also an emissary of an institution, and some accounts were plainly addressed past me to an imagined policy audience. The community dialogue was, in part, a way of surfacing and testing this. Leaders were given the emergent interpretations and invited to contest them, and their contestation is recorded in the analysis rather than smoothed away. The claim made here is therefore not that my position granted privileged access, but that it was one of the conditions under which this particular knowledge was produced, and that naming it is what standpoint theory requires (Haraway, 1988; Harding, 1996).

## Findings

Participants’ accounts are read here not as descriptions of wellbeing practices but as expressions of a migrant standpoint: a way of knowing wellbeing, distress, and care that is produced from a particular, migration-shaped position. Across the interviews, mental health was not defined in narrowly clinical or individualized terms but as a balance sustained across spiritual life, family, moral conduct, social legitimacy, and the capacity to meet everyday obligations; distress, correspondingly, was narrated through strained relationships, economic pressure, dislocation, racialized stress, and the weakening of communal supports. The analytic claim is not simply that participants held this relational understanding, but that their position let them see the individualized model built into Canadian services as one contingent framework among others, and to judge its limits from outside it.

Four interrelated dimensions of this standpoint emerged from the analysis. First, participants articulated a relational understanding of wellbeing that resisted individualist framing. Second, they exercised translated survival knowledge: strategic judgments about adaptation, concealment, and continuity across contexts. Third, they read institutional misrecognition from the inside, knowing formal systems through where those systems failed to grasp culturally grounded care and the meanings of distress. Fourth, they interpreted Alberta through a double vision, reading it not as a neutral setting but against other moral and social arrangements. What unites the four is not a catalogue of experiences but a single comparative capacity: migration made the assumptions of each social world visible from the vantage of another. This standpoint was internally differentiated, and the differentiation was patterned. Gender shaped who was expected to absorb the relational labour that sustained wellbeing and who was expected to provide materially, and so what counted as failure. Migration pathway shaped exposure to institutional scrutiny, with international students and those on precarious permits describing a vigilance around disclosure that citizens did not. Occupational location, and credential devaluation in particular, shaped whether institutions were read as indifferent or hostile. Religious affiliation shaped whether distress was narrated as spiritual, social, or clinical, and which help was available without stigma. Length of residence shaped the sharpness of the comparative vision, most acute among the recently arrived and, for the longer-settled, a more resigned double consciousness. Where these differences bear on the interpretation, they are marked below.

### Migrant standpoint as relational wellbeing

Participants consistently understood wellbeing in relational rather than purely individual terms. To be mentally well was not simply to feel calm, happy, or symptom-free. It was to remain connected, balanced, and socially anchored across one’s spiritual life, family relationships, community ties, and everyday responsibilities. In this framing, wellbeing was not treated as a private inner state detached from social life. It was understood as something sustained through connection to what is meaningful, morally grounding, and collectively supportive. As one participant explained,

“So mental health means feeling well, thinking well, doing well, being well, experiencing life. Well, when you’re in down periods, you have to be able be connected to something important to you… I think the sense of togetherness and unity and also too ‘Yɛ ka ne sɛn? Nkogya nkogya, ɛno yɛ proverb’ so life is like that. Wherever we go, we try to form a community and get ourselves known to help each other and support each other” (EA, man, 2 years in Canada).

Another participant similarly emphasized that wellbeing could not be reduced to whether immediate practical needs were met, noting that wellbeing “is not really about what’s happening in your day-to-day because my basic needs were met, but … I had to connect with my spiritual side” (AN, woman, 20 + years in Canada).

Read as standpoint, these accounts do more than locate wellbeing in social and spiritual ecologies rather than individual emotional states. They register a position from which the individualized model can be seen as partial, a way of knowing that life feel coherent, bearable, and worth sustaining.

This framing also tied wellbeing to dignity, moral order, and the ability to meet expected roles within networks of care and accountability. Participants’ accounts suggested that distress was not only a matter of internal difficulty, but could also arise when culturally meaningful ways of interpreting and responding to suffering were displaced or ignored. This was evident in the leadership dialogue, where one participant reflected on the limits of dominant institutional responses:

“I do think that when we realize that it is mental health and those institutions oftentimes could be walking a thin line where it’s actually backfiring instead of supporting. So what I mean by that… How does our culture view mental health to begin with? So there’s someone who’s clearly struggling with mental health issues in our community, they will say all these sort of things, and the moment we categorize it or we view it through our cultural lens, then the solution is not medication or the hospital or those institutions within society” (RO, leadership conversation).

What participants reveal here is an important dimension of migrant standpoint. Migration did not erase prior understandings of what it means to live well. Instead, it placed those understandings in tension with a setting in which communal density, time availability, economic pressure, and institutional norms often differed sharply from more familiar social worlds. Participants were therefore engaged in ongoing interpretation of how relational wellbeing could be maintained under conditions that more often rewarded individual self-management than collective interdependence. Their accounts reveal not only cultural differences but also a migration-shaped way of seeing the social conditions under which wellbeing becomes possible, fragile, or misrecognized. That interpretation was also gendered, and saying so is not an afterthought. Intersectionality was developed in the first instance to nuance a gendered analysis, and relational wellbeing is not a gender-neutral achievement. The relational ties that participants named as the substance of wellbeing, namely feeding people, hosting them, attending their funerals, checking on the isolated, holding the church group together, are ties that somebody has to maintain, and in participants’ accounts that somebody was overwhelmingly a woman. Migration intensified this rather than relieving it. In Alberta, the aunts, sisters, and neighbours who had distributed that labour in Ghana were absent, while the expectation that it be performed was not. Women therefore described a specific form of distress that men did not: not the absence of community, but the exhausting obligation to manufacture it single-handedly while also being a full-time student.

“I figured out how to, like, time manager myself better, so when I get home, I can have enough time to study, take care of the children and also get enough rest. And just balance my school and home life well. I’m only able to do it now as I progress through school, I’m finding ways to manage it better” (AE, woman, 5 + years in Canada).

Men, correspondingly, described a distress organized around provision and status, in which failure to earn, to remit, or to convert Ghanaian credentials into Canadian employment registered as a failure of personhood. Relational wellbeing, in short, is a shared value that is unequally paid for, and the migrant standpoint it generates is correspondingly gendered.

“They know it’s assured they are going to have a future. They have jobs waiting for them, they have those privileges. But for me, I have to be careful with that because if someone who lives here goes to look for a job sometimes and comparing with me to that person, they would sometimes consider that person more than they would to me.” (PQ, man, 2 years in Canada).

### Faith as infrastructure: religion and the authority to interpret distress

Religion recurred across the interviews with a consistency that resists treating it as one component of wellbeing among others. Participants prayed, attended church, sought pastoral counsel, and described spiritual grounding as the thing that held when other supports did not. Read only as content, this confirms an already familiar observation about religiosity among Ghanaian migrants. Read as an analytic category, it does something more consequential: it identifies the institution through which many participants exercised interpretive authority over their own distress. The church was where distress could be named without being pathologized, where it could be attributed to causes that Canadian mental health services do not recognize, and where the response to it was neither medication nor referral but prayer, fellowship, and the restoration of standing. AN’s account, above, makes the point precisely: material need had been met, and the deficit that remained was spiritual. No service framework organized around symptoms and social determinants can register that as a deficit at all.

This matters for the argument of this article because it locates a rival explanatory system, not merely a cultural preference. Ghanaian religious cosmologies supply their own aetiologies of misfortune, their own experts, their own remedies, and their own criteria for recovery, and they have institutional depth in the diaspora that secular community organizations rarely match (Read, 2019; Read et al., 2009). When participants engaged selectively with formal mental health services, they were frequently not choosing between help and no help. They were choosing between two systems that make incompatible claims about what has gone wrong and who is competent to say so. Selective engagement, read this way, is not avoidance born of stigma or low literacy. It is a considered judgment made from a position that has access to both systems and can see the limits of each, which is exactly what a standpoint, as distinct from a social location, consists of.

Religion also differentiated the sample internally rather than unifying it. Participants active in Pentecostal and charismatic congregations, which dominate the Ghanaian religious landscape in Alberta as in Ghana, described spiritual causation and spiritual remedy in the strongest terms and were most sceptical of psychiatric framing. Participants from mainline Protestant and Catholic backgrounds, and the smaller number of Muslim participants, tended toward accounts in which faith supported rather than replaced clinical help. Participants with weak or no religious attachment, a minority, were the most likely to describe the community’s religiosity itself as a barrier, since it left them without a legitimate vocabulary for distress at all. Gender cut across this: women described church as their principal source of practical support and their principal source of surveillance, since the same networks that provided childcare and food also carried judgments about whether a wife and mother was managing.

Treating religion as an analytic category rather than a descriptive attribute therefore does not produce a single religious migrant standpoint. It produces a map of the different positions from which distress can be authoritatively named within one migrant community (Collins, 2010; Yuval-Davis, 2006).

### Translated survival knowledge across everyday life and institutions

A second dimension of migrant standpoint emerged in participants’ accounts of adaptation, translation, and strategic adjustment. Participants often described migration as requiring continuous judgment about what to carry forward, what to modify, and what to withhold in order to remain functional and socially legible in Alberta. This was evident not only in major institutional encounters but also in the ordinary demands of daily life, where unfamiliar routines and norms had to be interpreted rather than simply accepted. One participant captured this through the seemingly mundane but revealing example of food:

“Because you are back home thinking, oh, when you come here you would adapt and stuff, but then you taste their food, and you are like, these combinations are not worth it… be it red pepper, yellow pepper, green pepper, but still no [hot] pepper, salts, and all those other seasoning. You cannot be like, nah, you can’t go this way… So yeah, I’m still taking time to adapt to their food and stuff” (PQ).

Trivial as it appears, the example marks migration as continuous interpretive labour rather than a single transition: a constant weighing of what to accommodate, what to refuse, and what to preserve to keep continuity with self and home.

Participants also described this labour as emotionally and socially demanding, particularly under conditions of isolation, uncertainty, and unmet expectations. Adaptation was not presented as passive acculturation, but as a repeated effort to make life workable when familiar supports were absent. As one participant explained,

“Okay, well, I’m a positive person. I’m very positive, open-minded person. But my stay here in Canada, I went through some difficulties like challenges, which it got to a time I felt like going back home because being in Canada alone with no family members around most especially you being an international student and not getting a job, the stress and how the depression that it is to take it quite difficult and very, very challenging, but all the same. I was like, oh, these were just temporary thing. It shall come to pass” (MG).

Others described learning to read the norms of work and social life through explicit comparison with Ghanaian expectations. As one participant noted,

“Yeah. So the work culture, the system here kind of values the individual’s output a lot more… In that sense you use that individual sort of acceptance and appreciation as compared to what we’ll call the Ghanaian kind of collectivist approach. Sort of was almost like a communal” (VZ).

These accounts are not reports of difficulty but demonstrations of knowledge: a migration-shaped capacity to interpret environments and compare social logics, and make strategic decisions about where flexibility was feasible and where maintaining continuity was essential for dignity and survival. What participants reveal here is a key dimension of migrant standpoint: translated survival knowledge, a socially learned capacity to read institutions, relationships, and risks from a position formed through movement across different social worlds.

### Institutional misrecognition, caution and selective engagement

Participants’ accounts highlighted forms of institutional misrecognition that shaped how support was pursued, trusted, or avoided. Formal resources were often recognized as present, but not always experienced as socially or culturally sufficient. Participants described a gap between the existence of services and the conditions that made those services meaningful, accessible, or necessary in the first place. One participant explained this contrast in terms of loneliness, social connection, and the limits of resource-based responses:

“So what I would say about that is yes, they do have more resources here. They’ll tell you if you’re experiencing this, you can call this line. Like oh, you’re experiencing this… people are going through all kinds of things but they don’t really know what to do. And I feel like yes, they do have more resources here, but then again people here are not as open, the culture, people don’t communicate as much, they don’t make friends as easily. So they have the resources. But then all in all, generally I feel like people in Ghana are more connected, they’re more friendly and social. So sometimes you may not even need to, don’t even get to the level where you start to experience mental health issues, because you feel lonely” (MW).

The account does not reject formal resources; it names a mismatch between institutional responses built around service provision and a wellbeing sustained through everyday social connectedness.

Participants also described selective engagement with formal systems in contexts where stigma, low awareness, and uncertainty about interpretation made help-seeking difficult. This was not simply a matter of refusing care. It reflected uneven recognition of distress and uncertainty about when, how, and on what terms mental health concerns could be named. As one participant noted,

“I feel like mental health is not preached a lot. And I feel like some people think it’s a weakness to sort of admit that you are going through something, especially when it’s a mental health thing. And because sometimes there’s that lack of awareness. People can be going through something and they don’t even know that they’re going through it. So maybe some people do know. So I think admitting is one thing, knowing in the first place one thing, but most of us who come here eventually get to know they preach it a lot all the time, every gathering you go to” (SS).

In the leadership dialogue, participants also pointed to more acute moments in which institutional systems failed to recognize culturally grounded forms of communication and de-escalation. One leader recounted an encounter in the emergency department:

“I rushed to the ED [emergency department], and there’s this patient being held upwardly by four cops or they were restrained. Our security guys couldn’t restrain them. Actually, what got me even more upset is they had to taser him inside a secure room. So they’re like, oh, he’s a trainer. He’s big. So I looked through and I see there’s a buffer guide, but he’s sitting there with all restraints and I look at the name and it’s a Nigerian name, right? And I was like, I realize you’re Nigerian. He goes, yeah, said, I’m from Ghana. And then a smile started breaking up, and then he stated …blah, blah, blah. They tased me. I’m like, are you hungry? I don’t even know why I said it. He said, yes. I said, I bet we could talk about Nigerian Jollof and Ghana jollof. I have some jollof with me. That was it. He was discharged the next day” (VA, leadership conversation).

What participants know here, and what the institutions serving them do not, is disclosed at the point of failure: they came to know these systems less through what was offered than through where recognition broke down around the social, cultural, and relational terms through which distress was experienced and responded to. What participants reveal here is a key dimension of migrant standpoint: knowledge generated through misfit. Repeated encounters with systems that demand legibility on unfamiliar terms sharpened participants’ sense of where trust was possible, where caution was necessary, and where parallel forms of support remained more viable.

### Double vision and comparative social knowledge

A further dimension of migrant standpoint emerged in participants’ comparative readings of life in Alberta. Participants often interpreted the Canadian context against other moral, social, and cultural reference points, including experiences in Ghana, memories of denser communal life, and expectations shaped through family and faith traditions. Alberta was therefore not encountered as self-evidently normal. It was read comparatively, sometimes as offering opportunity, but also as reorganizing responsibility, social connection, and the terms through which wellbeing and distress were understood. This comparative orientation shaped how participants judged parenting expectations, social boundaries, mental health definitions, and the kinds of support that were seen as possible or legitimate.

One participant illustrated this clearly in reflecting on differences in communal responsibility for children:

“When we differentiate ourselves from Western culture, if you bring forth a child, you are responsible for your child. No one cares about what your child is doing or even your wellbeing. But the Ghanaian community is in a way that if you need support, even our culture back home, yet a neighbour can hold your child accountable for anything. They see the child as their child. So they’re responsible for the child too. The system here, people are very careful and because of where we find ourselves now. There are some things he can’t do to a [neighbour’s] child, there are some things you can’t even say to their child” (AD). This account did more than describe cultural difference. It showed participants reading Alberta through an alternative moral framework in which care, accountability, and child-rearing were organized more collectively. A similar comparative logic appeared in participants’ reflections on mental health itself. As one participant explained,

“I think when you go back to Ghana and you see what they consider someone who is mentally unwell is someone who is maybe in the streets yelling, maybe bringing some sort of disgrace to their family, whereas one here who might be considered mentally unwell is still functioning. They’re still going to work. They still have a wife and kids, they still have family. They’re still able to drive and do all the things that they do. I think that because the definitions are so different, and at the end of the day, can you blame them? That’s just how they came up as what mental unwellness is, what mental health is that’s ingrained in them” (AB).

This is the comparative move in its clearest form: not a contrast between Ghana and Canada but knowledge generated by movement, in which the assumptions of each setting become visible only from the vantage of the other, including what counts as distress, what becomes recognizable as suffering, and when intervention is thought to be necessary.

Together, these accounts reveal a key dimension of migrant standpoint: double vision as comparative social knowledge. Participants were not only adapting to Canadian institutions and norms. They were also evaluating them from positions informed by elsewhere, before, and otherwise. This comparative vantage allowed participants to identify features of Alberta life that may appear ordinary within dominant frameworks, including thinner community support, more privatized responsibility, and narrower or differently calibrated understandings of distress. What participants reveal here is not nostalgia or simple contrast. It is a migration-shaped way of seeing in which movement across worlds makes social norms more visible, more contingent, and more open to critique.

## Discussion

This article has argued that migrant standpoint is a useful sociological concept for extending intersectional migration scholarship. Intersectionality remains indispensable for showing how migrant lives are organized through co-constituted relations of race, gender, class, migration history, culture, and other structures of power (Anthias, 2008; Yuval-Davis, 2006). However, it is often less explicit about how these locations become sites of interpretation, judgment, and critique, even though standpoint scholarship suggests that socially situated positions can also become grounds for knowledge production (Harding, 1996; Rolin, 2009). The findings presented here suggest that migration does more than expose people to inequality under new conditions. It also shapes epistemic orientation, that is, how social life is interpreted from positions formed through movement, settlement, racialization, and negotiation with unfamiliar institutions. Through participants’ accounts of relational wellbeing, translated survival knowledge, institutional misrecognition, and double vision, this study shows that migration can generate socially situated ways of knowing that are neither reducible to identity category alone nor fully captured by structural description. In this sense, migrant standpoint names not a universal migrant consciousness, but an analytically identifiable mode of knowing that emerges when migration is treated as a constitutive social process rather than as background context (Amelina, 2022; Anthias, 2008).

These findings are unintelligible without the society participants came from, and reading them only against Canadian migration scholarship reproduces precisely the epistemic asymmetry this article sets out to contest. Participants did not invent a relational conception of wellbeing on arrival in Alberta. They brought one. Gyekye’s (1996, 1997) account of Akan and broader Ghanaian moral thought describes a communitarian conception of personhood in which the individual is constituted through, rather than prior to, relationships of obligation, and in which a person’s wellbeing is a function of their standing within a web of kin and community duties. On this account, to be well is to be in right relation, and to be unwell is, in significant part, to have fallen out of it. Gyekye is careful to insist that this communitarianism is moderate rather than absolute, preserving individual agency within a relational frame, and participants’ accounts bore that nuance out: they neither dissolved themselves into the collective nor claimed the unencumbered autonomy that Canadian institutions appeared to presume of them (Gyekye, 1996, 1997).

The same holds for how distress was interpreted. Ghanaian mental health scholarship has documented a plural landscape in which biomedical psychiatry coexists with, and is frequently subordinate to, religious and traditional healing; in which severe mental illness is understood through spiritual and moral causation as much as through pathology; in which families rather than services carry the burden of care; and in which formal psychiatric provision is scarce, concentrated in a few urban institutions, and widely distrusted (Ofori-Atta et al., 2010; Read, 2019; Read et al., 2009). Crucially, this literature also shows that the threshold for recognizing someone as mentally unwell sits at a very different place than in Canadian clinical practice: it attaches to visible breakdown of social functioning and of the capacity to fulfil adult roles, rather than to internal symptom states. This is exactly the distinction AB draws in the passage above, and it explains why so many participants could describe Canadian definitions of mental illness as capturing people who, by the standards they grew up with, are manifestly coping. Participants were not failing to understand Canadian mental health categories. They were applying a different and internally coherent set of them.

Recognizing this changes what migrant standpoint is a standpoint on. The comparative vision described in the findings is not a vague sense of cultural difference. It is the operation of a determinate alternative logic, carried from a society with its own well-documented conceptions of personhood, affliction, and care, and brought into contact with institutions that neither recognize it nor know that they are failing to. That contact is what makes the assumptions of the Canadian system visible to participants and invisible to the system itself. Migrants are, in this precise sense, better positioned than the institutions that serve them to describe what those institutions take for granted, which is the epistemic advantage that standpoint theory has always claimed for subordinated positions (Harding, 1996; Rolin, 2009).

This is also where migrant standpoint both draws on and departs from institutional ethnography. With Smith (1987, 1992), the analysis begins from the everyday worlds of people positioned outside the centres of institutional authority and treats their accounts as an empirical entry point into the institutional relations that organize those worlds; the findings on institutional misrecognition and selective engagement are, in this sense, an account of how Canadian mental health and settlement institutions are put together as seen from below. Where the present concept extends that tradition is in the source of the critical purchase. For institutional ethnography, the standpoint’s analytic leverage comes from its location within a single ruling order whose coordinating relations it renders visible. For migrant standpoint, the leverage comes additionally from the participant’s prior socialization in a different institutional and moral order, which supplies an external vantage from which the host order’s arrangements appear as one possibility among others rather than as the natural state of things. Migrant standpoint thus retains institutional ethnography’s commitment to beginning from lived experience and reading institutions from that beginning, while adding a comparative, cross-institutional dimension that the single-order framing of classical institutional ethnography does not foreground (Smith, 1987, 1992).

It is worth being explicit about the limits of this affinity, because the differences are as instructive as the shared point of departure. Migrant standpoint belongs to the tradition of feminist standpoint epistemology associated with Harding and Haraway rather than to institutional ethnography as Smith and Griffith (2022) set it out, and it does not take up several of institutional ethnography’s defining commitments. It does not treat the researcher’s own location as the foundational ground of inquiry; positionality matters here, and is reported at length, but as one of the conditions under which situated knowledge is produced rather than as the method’s point of origin. It does not set out to map the textually mediated ruling relations that coordinate action across sites, or to “talk back” to those relations as institutional ethnography does; its object is the comparative interpretive capacity that migration generates, not the institutional circuits through which governance is organized. And it does not share institutional ethnography’s refusal of thematic coding or its self-understanding as a project at odds with standard sociology. The present analysis codes interview and dialogue material into analytically organized dimensions and offers migrant standpoint as a concept for use within intersectional and standpoint sociology, not as a break from it. The purpose of naming these differences is not to keep institutional ethnography at arm’s length but to specify what kind of concept migrant standpoint is: an epistemic claim about how migration-shaped positions become grounds for knowing, offered in the feminist standpoint tradition, that shares institutional ethnography’s commitment to beginning from lived experience while pursuing it by different means and toward different ends (Harding, 1996; Haraway, 1988; Smith and Griffith, 2022).

The sociological value of this concept lies in its ability to connect position, process, and knowledge. Participants were not only differently located within Canadian social life. They also interpreted that life through comparative and relational frames shaped by prior moral worlds, altered support structures, and repeated encounters with institutions that did not always recognize their terms of wellbeing and distress. This matters because it shifts analysis beyond the familiar claim that migrants experience layered disadvantage. It shows, more specifically, how migration-shaped lives can produce critical social knowledge about what dominant settings normalize, overlook, or misrecognize. Migrant standpoint therefore matters sociologically because it draws attention to migrants not only as subjects of inequality but as interpreters of social order, a perception this study locates in the fragility of social connectedness, the privatization of responsibility, and the unequal fit between dominant service frameworks and culturally grounded care (Anthias, 2002, 2008; Collins, 2000; Yuval-Davis, 2006).

The paper also contributes to sociological work on health and migration by showing that mental health promotion is not merely a technical matter of service delivery, literacy, or prevention messaging. It is a social field in which recognition, moral legitimacy, belonging, and institutional fit are actively negotiated (Jarvis and Kirmayer, 2023; Kirkbride et al., 2024). Mental health promotion is a revealing analytic site precisely because it makes visible the friction between dominant institutional models and migrants’ lived frameworks of care. It also shows that the promotion of wellbeing depends not only on access to services, but on whether the surrounding social and institutional environment can recognize and support the relational, moral, and culturally mediated conditions through which life remains bearable and meaningful (Compton and Shim, 2015; Olanlesi-Aliu et al., 2025; Patel, 2005).

It is fair to ask what migrant standpoint accomplishes that existing concepts do not, since each of the four dimensions reported here could, taken alone, be accommodated by an established framework. Relational wellbeing could be read as acculturation research on collectivist and individualist value orientations. Translated survival knowledge resembles what the literature on migrant navigational capital and everyday resilience already describes. Institutional misrecognition is legible as cultural competence failure or as a barrier to access. Double vision is a familiar cousin of Du Boisian double consciousness and of Anthias (2002, 2008) translocational positionality. The concept must therefore earn its keep, and it does so on three grounds rather than by renaming what is already known.

First, the four dimensions are related, not merely co-present, and the relation is what the existing frameworks miss. The comparative vision that let AB see Canadian and Ghanaian thresholds of unwellness as equally contingent is the same capacity that let MW judge that Alberta’s abundant crisis lines address a problem that denser sociality would have prevented; and that judgment is what made selective engagement rational rather than avoidant. Acculturation frameworks treat these as separate variables: value orientation, coping, service use. Migrant standpoint treats them as a single interpretive apparatus, and predicts, as those frameworks do not, that they will move together. Second, the concept is epistemic rather than descriptive. Cultural competence literature asks how services can better accommodate migrants’ beliefs, which concedes in advance that the service holds the valid account and the migrant holds a belief. Migrant standpoint asks what migrants know that services do not, and treats participants’ accounts as knowledge claims with truth conditions, some of which the institutions in question are poorly placed to assess. This is a different relation between researcher and account, and it yields different findings from the same data.

Third, and most importantly, migrant standpoint completes a move that intersectionality begins but does not finish. Translocational positionality gives an exemplary account of how migrant positions are constituted across shifting relations of time, place, and power, but it remains an account of position. Collins (2000, 2010) outsider within comes closest to what is needed, and this article is frankly indebted to it, but it was developed to theorize marginal proximity within a single national social order, and it does not address what happens when the outsider carries a rival social order with them and can therefore see the host order as one arrangement among possible others. That is the specific difference. Migration does not only place people at the margin of a structure; it gives them a second structure against which to read the first. The four dimensions reported here are evidence of a new concept precisely because they cohere as the exercise of that comparative epistemic capacity, and no existing framework in the migration literature names it as such (Amelina, 2022; Amelung et al., 2024).

A boundary question follows from this. If migration supplies the second order against which the host order is read, what of participants who did not themselves migrate? Three interviewees were born in Canada to Ghanaian parents, and their accounts caution against treating personal border crossing as the necessary condition of a migrant standpoint. What the concept requires is not the biographical fact of migration but sustained socialization within a diasporic moral and institutional order that differs from the dominant one, together with repeated encounters in which the two are brought into contact. That socialization can be transmitted across a generation through family, language, religious community, and association, so that second-generation participants may hold the comparative vantage without having crossed a border to acquire it. Read this way, migrant standpoint is better understood as diasporically rather than strictly migrationally constituted, and the Canadian-born participants mark not an anomaly in the sample but the transmissibility of the standpoint itself (Anthias, 2008; Yuval-Davis, 2006).

### Implications for sociology and migration scholarship

The concept of migrant standpoint has two implications for sociology and migration scholarship. First, it shifts analysis from migrant position alone to migrant knowledge production. Rather than asking only how migrants are unequally incorporated, it asks what becomes visible from lives shaped by movement, comparison, and institutional negotiation. This opens a more interpretive sociology of migration, one attentive to how assumptions about care, family, autonomy, belonging, and legitimacy are rendered visible from migration-shaped social locations.

Second, migrant standpoint challenges scholarship that treats migrants primarily as populations to be measured, integrated, or managed. The findings presented here instead show migrants as interpreters of institutions, producers of practical social knowledge, and critics of social and institutional misfit. This does not weaken structural analysis. It deepens it by showing how inequality is not only experienced, but also read, judged, and navigated from within migration-shaped lives. Migrant standpoint, therefore, preserves the power analysis of intersectionality while extending migration scholarship toward a fuller account of lived epistemology.

## Limitations

Three boundaries of this study should be stated, and stated as boundaries rather than as apologies. This article develops a concept through one case, namely Ghanaian accounts of mental health promotion in Alberta, and the warrant for the concept is analytic rather than distributional. The question is not whether twenty-one people represent a population, a criterion imported from a form of inference this study is not making, but whether the case is rich enough to specify the mechanism and disciplined enough to constrain it. What the case cannot do is tell us how that mechanism varies. Ghanaian communities in Ontario, with six decades of institutional density behind them, or Ghanaian communities in settings with different racial formations, legal regimes, or religious landscapes, may well generate configurations of standpoint that this study cannot anticipate. Establishing that variation is a task for comparative work, and it is the most obvious next step.

The second boundary concerns the sample itself. Recruitment proceeded through community leaders, associations, and churches, which is consistent with the study’s relational commitments but which systematically privileges those who are already connected. Ghanaians in Alberta who are estranged from the community, who are not religiously affiliated, who are undocumented, or whose distress has already isolated them are precisely the people least likely to be reached this way, and their absence should be read into the strongly relational account of wellbeing reported here. A standpoint constituted through community may look different, and considerably bleaker, from outside it.

The third concerns the analysis rather than the data. Although the sample spanned varied migration pathways, ages, occupations, and lengths of residence, the depth of the intersectional analysis is uneven across those axes. Gender and religion are developed substantively above; class, and in particular the experience of credential devaluation among participants whose professional qualifications were not recognized in Canada, is identified but not fully worked through, and generation and legal precarity are touched on only lightly. These are not defects of the concept but limits of what a single study can carry, and they mark where migrant standpoint most needs to be tested next.

## Conclusion

This article has proposed migrant standpoint as a useful extension of intersectional migration scholarship. Drawing on Ghanaian experiences of mental health promotion in Alberta, it has argued that migration does more than intensify or rearrange social inequality. It also generates socially situated ways of knowing through which wellbeing, care, belonging, and structural constraint are interpreted. Participants’ accounts showed that these ways of knowing emerged through relational obligation, strategic adaptation, institutional misrecognition, and comparative evaluation across social worlds. Taken together, the findings suggest that migrants are not only differently positioned within unequal social relations. They are also interpreters of the social worlds they inhabit and navigate.

The value of migrant standpoint lies in its ability to connect structure, movement, and knowledge without reducing migrants to either victims of inequality or uncomplicated agents of resilience. It keeps power in view while recognizing that migration-shaped lives can generate critical insight into the conditions under which wellbeing becomes possible, fragile, or misrecognized. In the context of mental health promotion, this matters because dominant frameworks often overlook what migrants already know about sustaining life under strain and about the institutional arrangements that support or undermine meaningful care. A sociology attentive to migrant standpoint is therefore better equipped to understand migrants not only as subjects of movement and inequality, but also as producers of knowledge about the social worlds they cross, inhabit, and remake.

## Data Availability

The raw data supporting the conclusions of this article will be made available by the authors, without undue reservation.
